# Supplementary material for: Delivery Capacity and Anticancer Ability of the Berberine-Loaded Gold Nanoparticles to Promote the Apoptosis Effect in Breast Cancer
Source: Cancers (Basel). 2021 Oct 22;13(21):5317. doi: 10.3390/cancers13215317 (PMC8582582; doi:10.3390/cancers13215317)
Supplement: Supplementary file 1 [file cancers-13-05317-s001.zip › cancers-1372113-supplementary.pdf]

Supplementary Materials: Delivery Capacity and Anticancer Ability of the Berberine-Loaded Gold Nanoparticles to Promote the Apoptosis Effect in Breast Cancer

Chen-Feng Chiu, Ru-Huei Fu, Shan-hui Hsu, Yang-Hao (Alex) Yu, Shun-Fa Yang, Thomas Chang-Yao Tsao, Kai-Bo Chang, Chun-An Yeh, Cheng-Ming Tang, Sheng-Chu Huang and Huey-Shan Hung [3,4,]

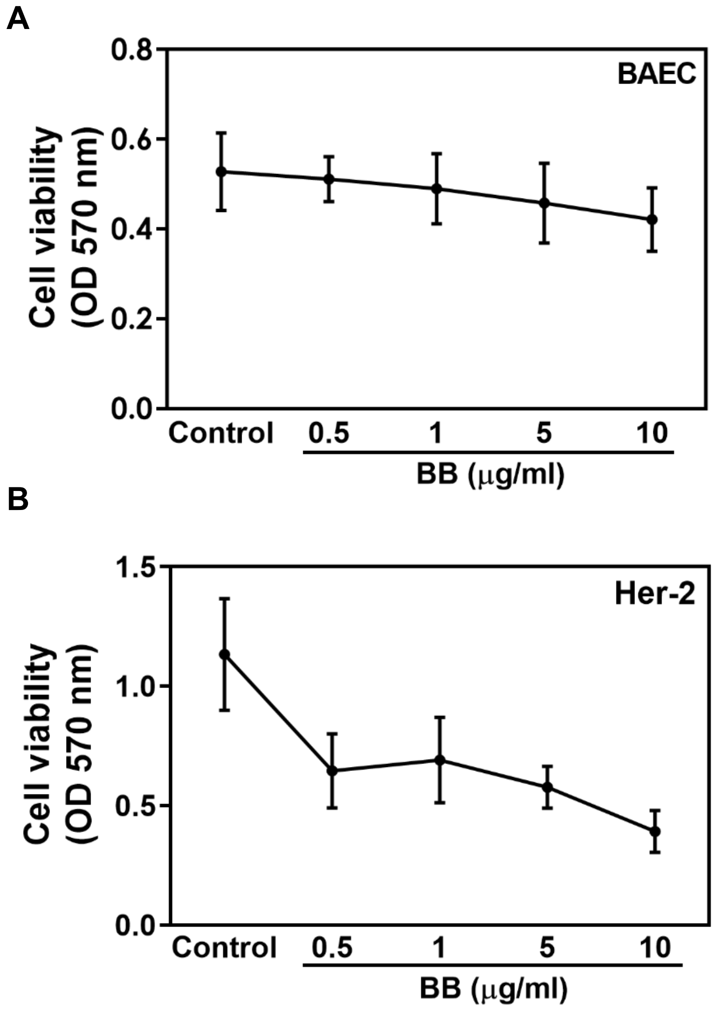

Figure S1. Comparison of cell viability between **(A)** BAEC and **(B)** Her-2 cell line seeded on various materials was examined by MTT assay and the results were quantified based on fluorescence intensity, showing that BB could significantly inhibit cell viability in Her-2 breast cancer cell line.

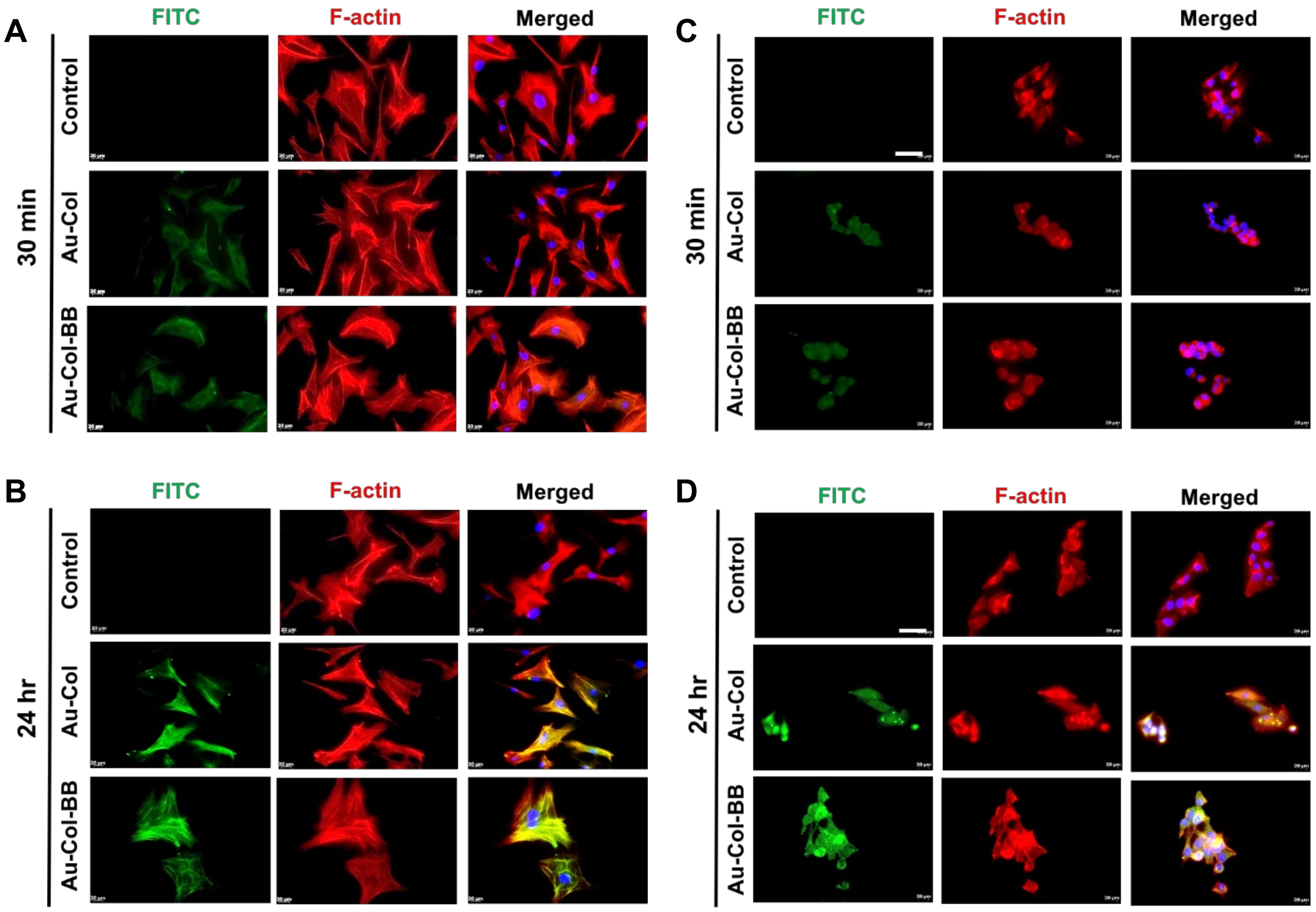

Figure S2. Assessment of cell uptake ability in BAEC and Her-2 cell line. Au-Col and Au-Col-BB were firstly conjugated with fluorescent dye (FITC) to investigate inside cell transportation, then observed by using fluorescent microscopy. The Au-Col and Au-Col-BB flagged with FITC were observed intracellularly in (A) 30 min and (B) 24 hrs of BAEC and in Her-2 breast cancer cell line (C) 30 min and (D) 24 hr. The green fluorescent amount was also determined by immunofluorescence intensity. Scale bars = 20 µm.

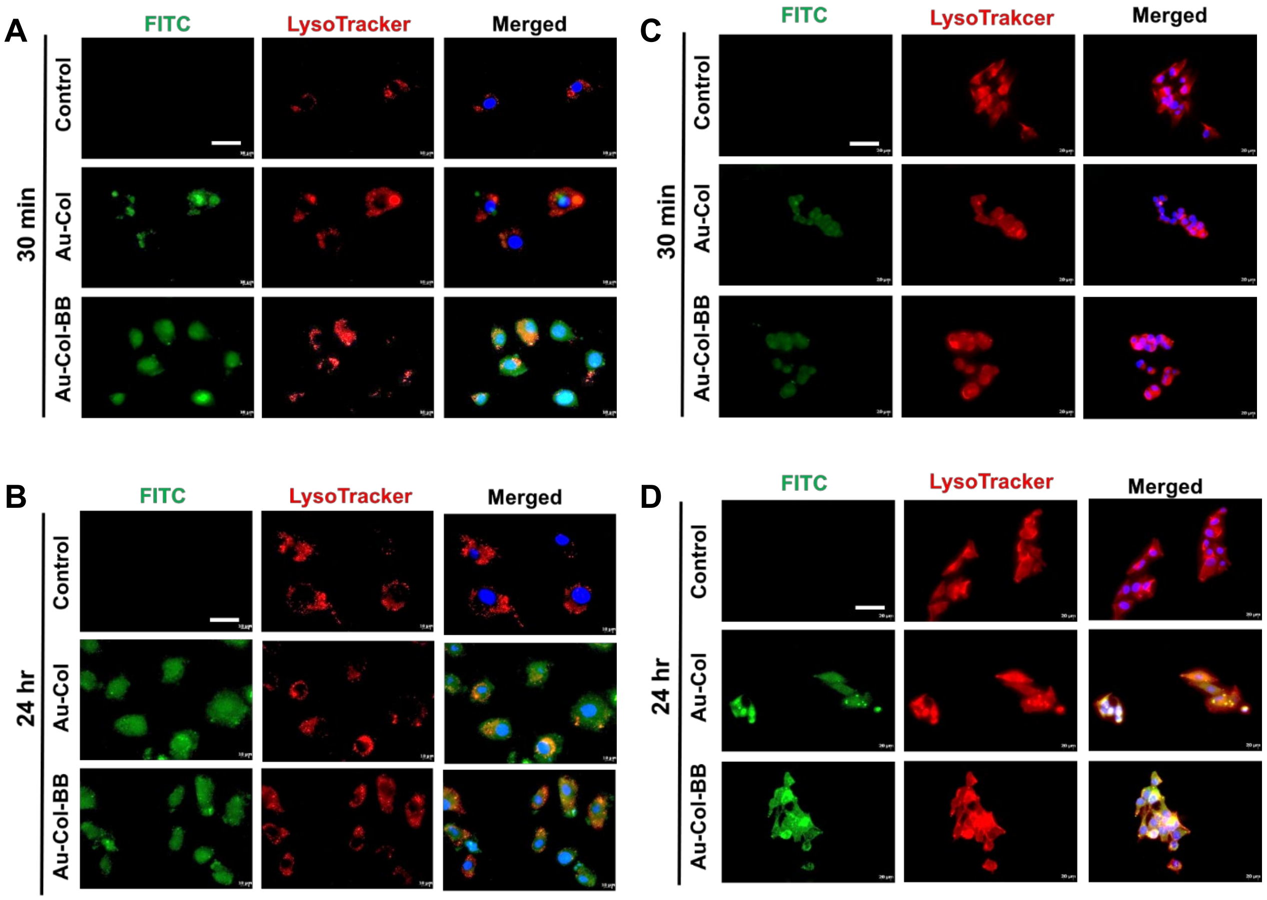

Figure S3. Evaluation of cell uptake ability in BAEC and Her-2 cell line by using LysoTracker. A red fluorescent probe, LysoTracker was applied to observe lysosomes so as to verify potential transportation ability, and the lysosomes were observed within cytoplasm, particularly perinuclear site. Assessment of cell uptake ability in Her-2 cell line. Au-Col and Au-Col-BB were firstly conjugated with fluorescent dye (FITC) to investigate inside cell transportation, then observed by using fluorescent microscopy. The Au-Col and Au-Col-BB flagged with FITC were observed intracellularly in (A) 30 min and (B) 24 hrs of BAEC and in Her-2 breast cancer cell line (C) 30 min and (D) 24 hr. The green fluorescent amount was also determined by immunofluorescence intensity. Scale bars = 20 μm.

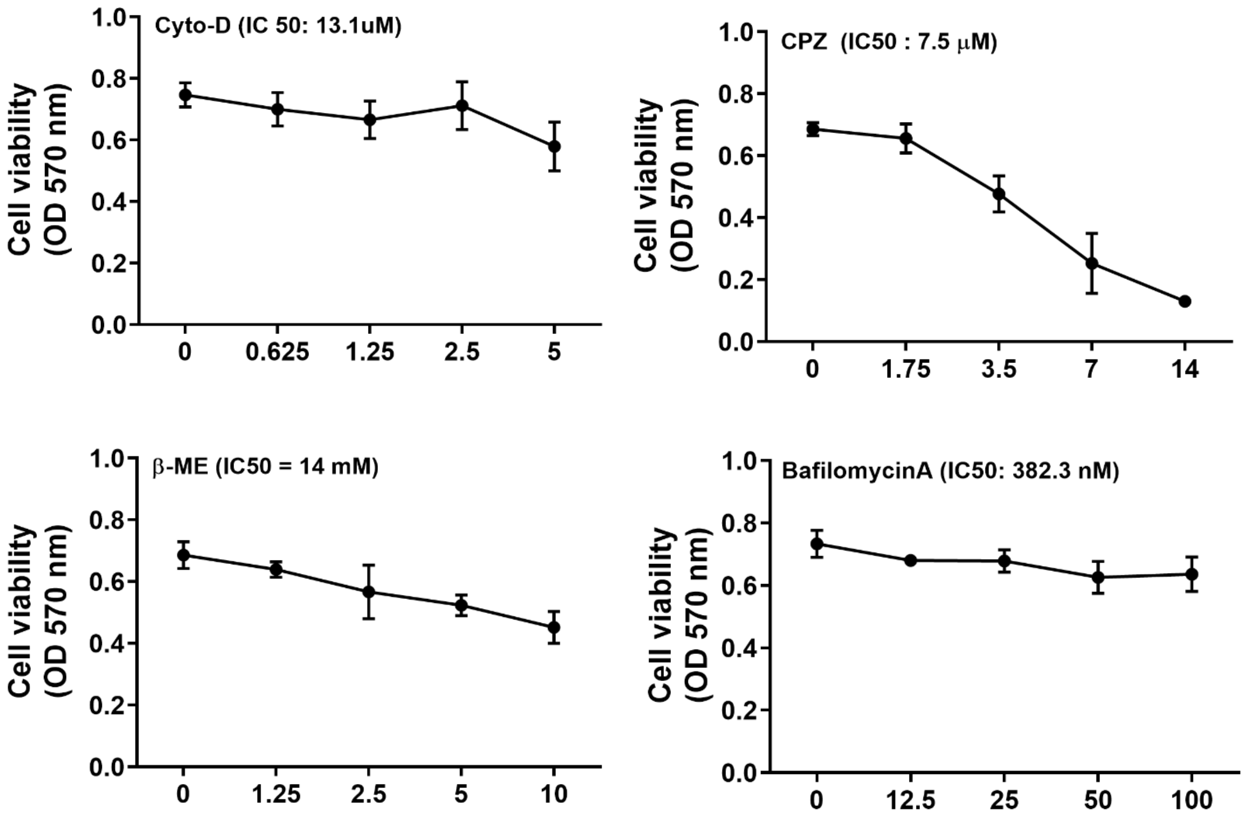

Figure S4. The concentration of four endocytosis inhibitors that caused 50% of cell growth inhibition. The results were presented as IC_{50}. The IC_{50} values represented as the concentration that cause 50% of cell growth inhibition evaluated by using 95% confidence intervals (CI 95%) from non-linear regression after normalize the results compared to untreated control group. Each experiment was triplicated. MTT assay were used to measure the 50% inhibitory concentration (IC_{50}): Cyto-D (13.1 μM), CPZ (7.5 μM), β-ME (14 μM), and Baf (382.3 nM).

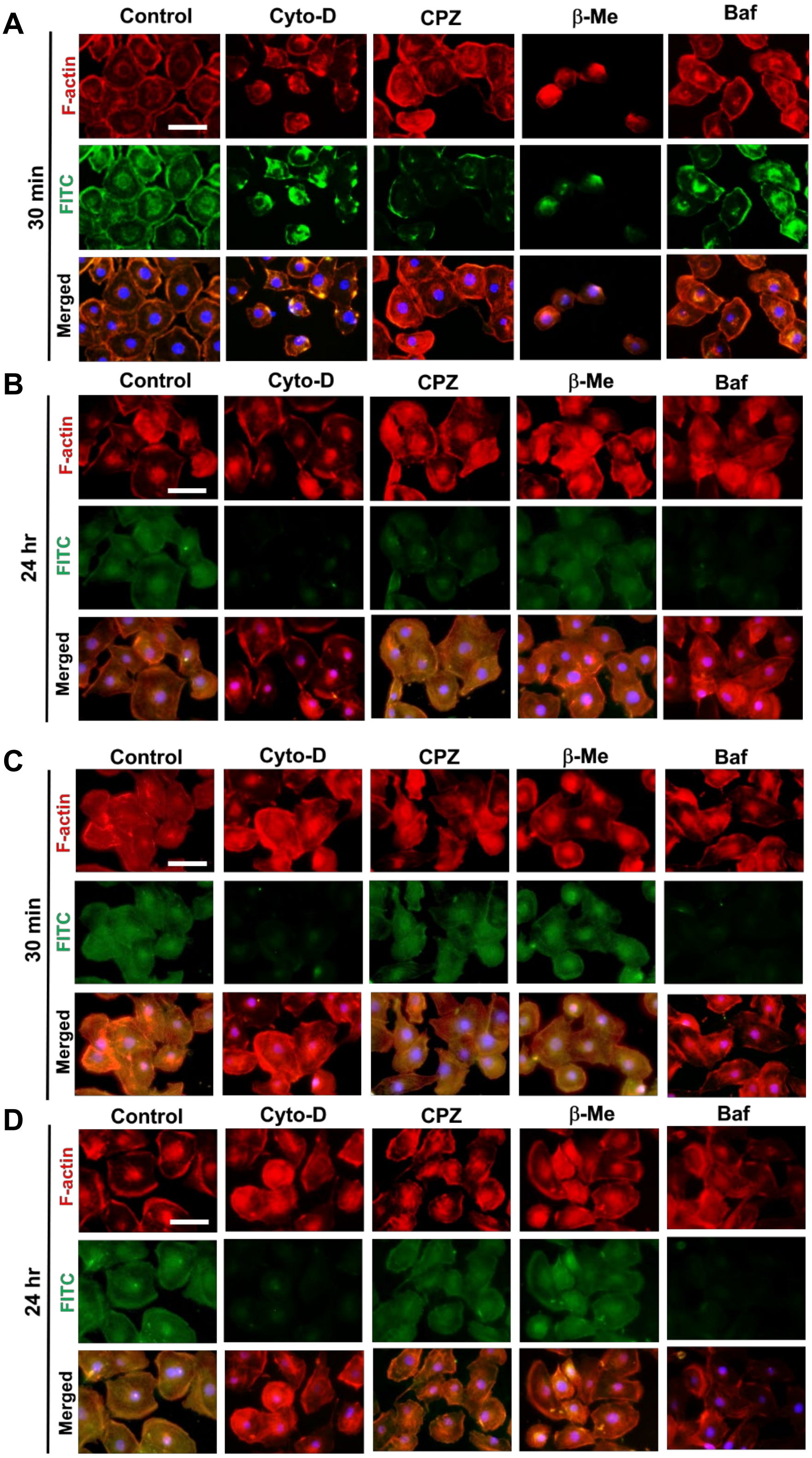

Figure S5. Assessment of endocytotic route for BAEC and Her-2 cell lines were also treated with 4 kinds of endocytosis inhibitors. There are several different types of endocytotic pathways: caveolae, macropinocytosis, receptor-mediated endocytosis, and phagocytosis. 4 different endocytotic inhibitors, Cytochalasin D (Cyto-D), Chlorpromazine (CPZ), 2-Mercaptoethanol (β-ME) and Bafilomycin (Baf) were incubated with Au-Col-FITC-BB (10 μg/ml) Assessment of cell uptake ability in Her-2 cell line. Au-Col and Au-Col-BB were firstly conjugated with fluorescent dye (FITC) to investigate inside cell transportation, then observed by using fluorescent microscopy. The Au-Col and Au-Col-BB flagged with FITC were observed intracellularly in (A) 30 min and (B) 24 hrs of BAEC and in Her-2 breast cancer cell line (C) 30 min and (D) 24 hr. The green fluorescent amount was also determined by immunofluorescence intensity. Scale bars = 20 μm.
